# Platelet-rich plasma as a site-targeted approach in wound healing: a molecular perspective

**DOI:** 10.15190/d.2018.8

**Published:** 2018-12-31

**Authors:** Teodora Veronica Grigore, Christian Cozma

**Affiliations:** Department of Biochemistry and Molecular Biology, University of Bucharest, Bucharest, Romania

**Keywords:** Wound healing, platelet-rich plasma, skin wound, skin regeneration, clinical trials.

## Abstract

Wound healing remained an equation with multiple variables that experts in the medical field are trying to solve. The need to find an adjuvant that can quicken the healing process is increasing with every day, as longer wound healing times raise the risk of infections. Platelet-rich plasma is a promising tool promoting faster healing in a variety of wounds (thermal wounds, burn wounds, surgeries, etc.), as a series of studies present encouraging results in patients that received platelet-rich plasma treatment. The aim of this paper is to review and comment on the useful benefits and limitations of using platelet-rich plasma as an adjuvant strategy in wound healing, emphasizing on skin related wounds.

## **1. Introduction**

Skin tissue regeneration and a fast regeneration are important aspects in achieving a proper wound heal, in order to avoid the risk of infections, bad scarring or death (in patients with pathologies that can affect the healing process). A quicker wound healing process also presents the advantages of being more cost effective, as the patient needs less nursing time.

The human skin is a three-layered organ: the epidermis (the surface of the skin), the dermis and the hypodermis (a subcutaneous adipose layer). The skin presents nerves, sensory corpuscles, vasculature and skin appendages, such as sebaceous glands, sweat glands and hair follicles. In the process of wound healing, the epidermis is the layer that is restored through the re-epithelialization process, while the dermis and the skin appendages help the re-epithelialization process through providing nutritional and mechanical support^1^.

The human epidermis consists of a stratified squamous epithelium that contains keratinocytes, melanocytes and no blood vessels. After an injury is inflicted, if the injured skin region fails to re-epithelialize, this leads to infections, losing the barrier function of the organ and even death. This urges the need to a rapid wound closure by the proliferation of epithelial cells in order to restore the barrier function that is critically important for survival^2^.

Currently, there are multiple strategies for skin tissue regeneration, such as split-thickness skin grafts, moisture dressing or the standard wound care. However, even if all these methods present valuable advantages, they also present disadvantages. For example, skin grafts can lead to the appearance of transplant rejection^3^, while moisture dressing is painful - not only applying it on the wound is hurtful, but it may sometimes also affect the nerve endings^4^.

Notably, a plethora of studies claim and appraise the beneficial effects of platelet-rich plasma (PRP) on cellular proliferation and tissue regeneration on the ground that its molecular components, such as growth factors or cytokines, play an important role in these processes. PRP represents the compound obtained through the separation of platelets from the other cellular populations present in a blood sample. PRP shows great promise in the field of skin wound healing as the molecules and cytokines found in PRP help stimulate collagen synthesis, extracellular matrix production and processes like angiogenesis or cellular proliferation.

The aim of this review is to highlight the benefits, efficacy and limitations of PRP use in wound healing.

## **2. Molecular factors correlated with wound healing timeline**

Wound healing naturally occurs after an injury and is an evolutionary conserved and complex process that requires an abundance of specific cells, growth factors and cytokines. These molecules form a signaling network that modulate a series of cells, including endothelial cells, fibroblasts, keratinocytes and immune cells, which are essential in wound inflammation and healing as they secrete many of the cytokines and growth factors. In these circumstances, the above mentioned cellular populations proliferate, migrate and achieve tissue repair^5^. The importance of wound healing stems from the fact that an untreated wound or a wound that has received improper treatment may aggravate through infections, Marjolin’s ulcer (rare and aggressive type of skin cancer that grows from burns^6^), other health complications or even death. These situations inconvenience especially patients that are suffering from thalassemia, anemia or diabetes, whose wound heal with difficulty compared to healthy patients, or their wounds may not close at all, depending on the size and depth of the wound.

**Figure 1:**
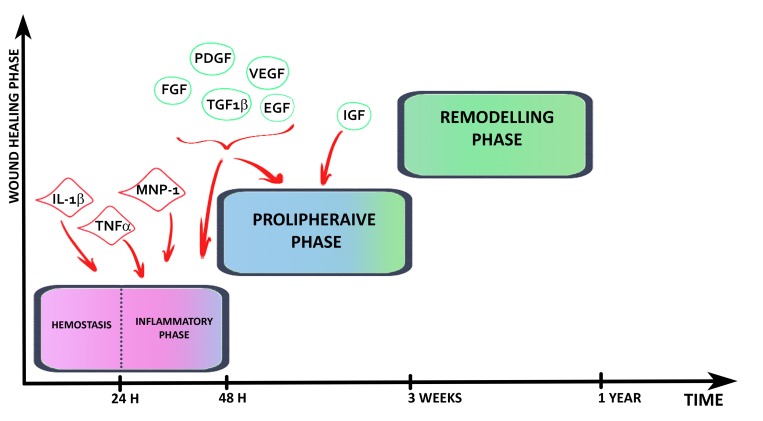
Wound healing phases and the molecules that are required for each phase

It is reported that wound healing has three main stages: the inflammatory phase, the proliferative phase and the remodeling phase. Conventionally, these stages are considered partly overlapping as a precise temporal delimitation is yet impossible to establish (see **Figure 1**).

The cellular response in the inflammatory phase is initiated right after the lesion is inflicted and can take up to 2 days. Some studies report that this phase actually starts only one day after the injury, while the first 24 hours are considered to represent the sub-phase known as hemostasis^7^.

The main target during the inflammatory phase is to sanitize the injury and remove foreign bodies in order to start the proper rebuilding of the skin tissue. In such manner, the first stage is marked by the formation of a blood clot through the aggregation of platelets and thrombocytes in a fibrin network^8^. The blood clot acts as a barrier against microorganisms and helps organize the temporary matrix that is needed for further cellular migration events^7^. This process is known as the hemostasis phase, being considered by some authors as a self-standing stage^9^. During this phase, molecules such as IL-1β, TNF-α, MNP-1, PDGF, FGF, VEGF and TGF-β1 are recruited at the lesion site for two main reasons. On one hand, they increase blood vessel permeability which eases tissue nutrition, while on the other hand, they are involved in the formation of the granulation tissue, which is necessary for the next stage’s evolution^7,10^.

The second stage, called the proliferative phase, is initiated 48 hours post-injury and lasts for up to three weeks. This phase is essential for the healing process, as it is marked by the formation of a viable epithelial barrier, allowing the wound to begin proper closing^8^. Granulation tissue may start forming the fourth day after the injury. This tissue’s formation is characterized by collagen synthesis and fibroblastic proliferation, essential for the development of any connective tissue. Angiogenesis is essential during this period, as proper vascular irrigation brings a fresh supply of oxygen and supplements to the cells^7^.

For this stage, growth factors such as PDGF, VEGF, FGF, IGF, or TGF-β1 are mobilized in order to start processes like angiogenesis or reepithelialisation^10^.

For instance, PDGF is mitogenic for fibroblasts and helps stimulate the fibroblast proliferation and migration, VEGF promotes angiogenesis, is a powerful mitogenic for keratinocytes or endothelial cells and helps mediate the extracellular matrix synthesis and deposition and FGF enhances the endothelial and fibroblast migration and proliferation, stimulates angiogenesis and is believed to help tissue repair through skin cells growth^11^.

TGF-β1 helps mediate extracellular matrix formation, helps the keratinocyte migration in re-epithelialization, stimulates type I and type III collagen production, angiogenesis and enhances the proliferation of fibroblasts. Regarding wound healing, IGF is believed to stimulate cell growth and proliferation^10^.

The third phase, named the remodeling phase, begins at the end of the proliferative stage and lasts for up to one year or even more. This phase can be defined as an attempt to recover skin tissue’s architecture, as the granulation tissue is remodeled. During the remodeling stage a variety of cellular events occur, such as emigration processes or apoptosis affecting the majority of fibroblasts, inflammatory cells or the endothelial cells. These processes may eventually lead to scar formation, which is characterized by a decrease in the number of cells^7^.

## **3. The mechanisms of tissue regeneration**

Tissue regeneration is a process that involves cellular renewal and regrowth, and it is essential in order to achieve a convenient and complete wound heal.

It is essential to mention that the length of each stage and the whole length of the healing process varies from individual to individual, and is influenced by endogenous and exogenous factors, such as diabetes, hereditary healing disorders, medication, smoking, alcoholism, nutrition, obesity and many others^7,9^.

The re-epithelialization of the wound accounts for 80% of the wound closure, and it depends on a series of factors such as the patient’s health conditions and genetics, as well as wound-related specifics: the location, the size, the depth or the contamination with microbes or bacteriae^12^.

The healing mechanism depends on the injury’s severity. If the wound is superficial and characterized by partial-thickness (only the epidermis and a small portion of the dermis are affected), it will heal mostly by re-epithelialization. However, if the wound is deep and described as having full-thickness (the epidermis and the dermis are destroyed), it will heal not only by re-epithelialization, but also through a granulation tissue formation in order to fill the gap inflicted. The main differences between a partial-thickness wound and a full-thickness wound are that the former lack the proper base which allows keratinocyte repopulation, and that, in the case remains of skin appendages, they do not regenerate until the complete dermis restoration^1^.

The re-epithelialization phase overlaps with the inflammatory phase, as the epithelial cells manifest a migratory activity at a few hours after the injury. In order to achieve the re-epithelialization of a partial-thickness wound, the distance needed to cover the wound is of approximately 500 µm, and, subsequently, in order to re-epithelialize a partial-thickness wound that has a 200 µm depth, a period of 8 days is needed in young human adults^1^.

As the re-epithelialization process is not possible without an intact dermis, the dermis structure should be further presented. The dermis consists of the papillary dermis (sub-epidermal layer) and reticular dermis, along with the dermal papillae, and the dermis consists of primarily fibroblasts and many matrix components, such as collagen, elastin or glycosaminoglycans. The different layers of the dermis have different healing capacities, with the papillary dermis that regenerates the fastest, followed by the mid-dermis and the lower reticular dermis^1^.

In order to restore the dermis, the void created by the wound is, as mentioned above, filled with a granulation tissue, that is deposited by fibroblasts, and it is presumed that, when the lesion is so strong that there are no fibroblasts left in the dermis, the granulation tissue is deposited by fibroblasts that belong to other regions and thus are imported. This granulation tissue consists in a population of macrophages, fibroblasts and a mix of collagen fibers, hyaluronic acid and fibronectin^1^.

Normally, hair follicle formation happens during embryonic development, but it has been observed that, at two or three days after the re-epithelialization of the wound, *de novo *hair follicles can be found in the regenerated area^2^.

## **4. Platelet-rich plasma (PRP)**

PRP is a plasma enriched with platelets that contain growth factors, that are considered to help with the processes of tissue regeneration and wound healing.

One of the many applications of PRP (and possibly the best known) is in dermatocosmetology, as PRP injections help restore skin’s youth through skin and neck rejuvenation, stimulating collagen synthesis^13,14^.

PRP is obtained through the concentration of platelets from a normal, whole-blood sample. There are different PRP processing systems available on the market: PRP kits that use specially designed columns, kits such as GloFinn PRP, XCELL PRP System or many other, and the platelet-pheresis procedure, but the centrifugation method is the most commonly used^15^.

Even though an exact protocol for processing and obtaining PRP has never been established, the basic steps are always similar: blood collection using anticoagulant followed by two consecutive centrifugations. Procedural differences concern the volume of anticoagulant that is used and centrifugation time and speed. Usually, two centrifugation steps are required: the first centrifugation separates the red blood cells, while the second centrifugation concentrates the platelets - the resulting product being platelet-rich plasma.

The use of an anticoagulant is essential, as this avoids the coagulation of the collected blood. There are different methods for using an anticoagulant – one distinct method is based on using EDTA-coated tubes, while other methods are based on collecting the blood in tubes that already have a specific volume of sodium citrate, acid citrate dextrose, calcium citrate, citric acid or citrate phosphate dextrose^16^.

Because it is believed that the anticoagulant can affect the release of growth factors and other molecules from the platelets, an activator is added in the sample, in order to stimulate the release of soluble molecules. Some studies report using calcium chloride, thrombin, type I collagen, calcium gluconate or even a mix of calcium chloride and thrombin, while other studies report skipping this step, as it is not entirely essential^17^.

PRP can be generated in different forms, each one presenting various advantages and disadvantages. Liquid PRP can be easily injected at target sites, thus being the most commonly used for PRP applications. Lyophilized PRP powder allows a better grasp of the growth factors concentration^18^, while gel preparation of PRP exhibits antiseptic properties^19^.

Usually, it is recommended to use different PRP quantities and types for each application, so the PRP used is custom made for an individual to serve a specific function. The demand for tailored/custom-made PRP applications stems from the physiological differences between individuals. The age, sex, and health condition of an individual affect the platelet count, which in turn directly alters the growth factors’ concentration^20^.

As a result of the diverse obtaining methods for PRP and taking into account the cellular content, there are different types of PRP that present different properties– leukocyte-poor PRP (LP-PRP), leukocyte-rich PRP or leukocyte-PRP (LR-PRP), pure PRP (P-PRP) and many others^21,22^.

Comparisons between LP-PRP and LR-PRP have been made, in order to elucidate the effect of leukocytes in PRP`s efficacy, and it has been demonstrated that the presence of leukocytes increases significantly the acute inflammatory response^23^ and causes a significant amount of cellular death if injected intra-articular, affecting especially synoviocytes^24^.

It is widely believed that PRP’s efficacy is due to the presence of growth factors and other molecules that are released by platelets. Some of the growth factors identified in PRP are the platelet-derived growth factor (PDGF), insulin-like growth factor 1 (IGF-1), vascular endothelial growth factor (VEGF), fibroblast growth factor (FGF), epidermal growth factor (EGF) and transforming growth factor β1 (TGFβ1). Other molecules that are found in PRP are tumor necrosis factor α (TNF-α), monocyte chemotactic protein 1 (MNP-1) and interleukins (IL), such as IL-1β, IL-5, IL-6, IL-8, IL-10, IL-1 receptor antagonist (RA), vitronectin, fibronectin and fibrin^25-27^.

PDGF has a powerful impact on wound healing, as it is implicated in angiogenesis, activation of macrophages and mitogenesis (being a strong mitogen for muscle cells and fibroblasts) and it is directly implicated in wound healing^28^. There are many isoforms for this thermo-resistant molecule, named PDGF-AA, PDGF-BB, PDGF-CC, PDGF-AB, and, more recently, PDGF-DD. PDGF also stimulates cell proliferation and cell growth and helps type I collagen synthesis^28,29^.

IGF is a growth factor that is believed to stimulate cell growth and cellular proliferation^10^. There are studies that claim that, when combined with PDGF, IGF can enhance the quality of wound healing^11^.

The VEGF family promotes angiogenesis by stimulating the proliferation of endothelial cells and their migration and plays a key role in vasculogenesis^29^. Two specific isoforms of VEGF, VEGF_164 _and VEGF_165_, have a key role in vascular development, but each isoform from the rest of the VEGF family has a different role arterial development and vascular patterning^30^.

FGF stimulates mitogenesis in chondrocyte populations, smooth muscle cells, osteoblasts, fibroblasts and skeletal myoblasts. Thereby, this growth factor plays a key role in the process of wound healing, as it promotes cell proliferation and tissue repair^29,31^. It has been shown that FGF promotes type II collagen production. However, it has no effect on the type I collagen biosynthesis^32^. Studies mention that the FGF expression peaks when an injury is caused, suggesting its importance in wound healing, especially in cutaneous wounds^33^.

EGF has been proven to have a mitogenic effect and to induce cellular migration on fibroblasts and epithelial cells^29^. EGF affects angiogenesis and tissue regeneration by having a role in the regulation of collagen secretion and cellular proliferation^10,34^. EGF stimulates wound healing through epidermal regeneration and by stimulating the proliferation of dermal fibroblasts and keratinocytes^11^.

TGF-β1 has multiple qualities, but the most important of all its attributes, it regulates the mitogenic effect of other growth factors. Among its characteristics count: the stimulation of angiogenesis, endothelial mitogenesis, extracellular matrix, type I and type III collagen production, it enhances keratinocyte migration in reepithelialisation^10,11,29^.

IL-1β, IL-6 and TNF-α are usually released in immune responses and have an important role in maintaining the homeostasis^35^. Studies show that cytokines can mediate processes such as immunological responses or cell-to-cell communication^36^. IL-6 plays a key role against infections caused through injuries and can ameliorate chronic inflammation^37^.

It has been observed that, while IL-1β cannot be found in uninjured skin, after a lesion is induced, a significant increase in this cytokine’s expression can be found in damaged tissues within the first 30 to 90 minutes. The same increase in expression was also observed for TNF-α, and IL-6 in the wound liquid, after a surgical intervention^38^.

Thus, PRP has growth factors that are important in diverse processes such as angiogenesis, mitogenesis or extracellular matrix and collagen production and thus has potential in cellular proliferation, tissue regeneration and wound healing.

## **5. The impact of PRP on wound healing**

As mentioned in the sections above, the possibility of an accelerated wound healing is crucial for a significant number of patients, which brings PRP into attention, as many researchers believe to quicken the process.

Currently, PRP is used worldwide as a skin youth rejuvenator, as it stimulates the type I collagen synthesis in the dermis, which allows the wrinkles to be partially or fully erased^39^. A number of studies suggest that PRP is able to stimulate the proliferation of dermal fibroblasts^40,41^, of keratinocytes^42^ and of endothelial cells^43^. It has also been shown that PRP may have a positive effect on epidermal regeneration, through the stimulation of cellular proliferation and migration, and also by bringing molecules involved in the process of wound healing at the lesions site^44^.

One experiment analyzed and compared three types of treatments for wounds in mice. The three groups were treated with PRP and human keratinocytes and fibroblasts, or with PRP and DMEM media, or with saline solution. It was observed that the group that received PRP and skin cells treatment completely healed wounds, while the other two groups did not achieve full closure of the wounds. It is mentioned that a fourth group of mice that had received only skin cells as treatment was excluded from the study, as the cells could not remain at the wound site and scattered through the encompassing tissue, proving that PRP has a positive effect on wound healing^45^.

Another application of PRP mixed with cells is the treatment with autologous PRP and adipose-derived stem cells (ASC). Researchers treated and analyzed the wounds of two groups, one group treated with standard wound care and the other group treated with autologous PRP and ASC. It has been observed that, while both groups had a high rate of wound closure, the group that received PRP treatment had a bigger rate of wound closure than the control group^46^.

The use of PRP with ASC is widely studied, as it is believed that, if cultured in keratinocyte differentiation media, ASCs differentiate in keratinocyte-like cells, which are required for proper wound healing^47^. Few *in vivo* studies with this combination are found in the literature, possibly as a result of the lack of a proper protocol. However, the existing *in vivo* studies show that patients that received PRP and ASC treatment presented a shorter time required for complete re-epithelialization of the wound and almost no recurrence of the chronic skin ulcer^48^.

An extensive analysis of studies regarding the efficacy of PRP treatment on wound healing after surgery showed that patients treated with PRP presented a significantly better vascularization through angiogenesis^49^, less pain^50^, and a 15.9 days difference between the control group and the PRP treated group in terms of healing time^51^.

Regarding the PRP treatment post-surgery, the use of PRP on skin flaps has proven that PRP stimulates angiogenesis through the activity of growth factors present in its composition (especially VEGF), thus further increasing flap healing^52^.

It has been observed, on two identical wounds induced on the same patient, that the lesion treated with PRP healed faster and left almost no scar tissue after 6 months compared to the lesion treated with thrombin. At six days after the incisions, the wound treated with thrombin developed an erythema and did not present an epithelial layer, while the wound that received PRP treatment was healthy and was covered in a thin epithelial layer^25^.

A PRP gel was applied to the wounds of 100 thalassemia patients (wounds such as burns or cuts), and it has been noticed that the wounds had a much shorter healing time and the lesions did not reopen while the 8 months that the patients were under surveillance^16^. Another study concluded that PRP treatment helps the healing time of the wounds regardless if the wound was caused by burn, cut, etc.^53^.

In order to assess the effect of PRP concerning the healing of burns, three groups of rats were compared, one group of rats with deep second-degree burns, one group of rats with deep second-degree burns associated with diabetes mellitus, and one group of rats with third degree burns. Each group was divided into a control subgroup and a PRP-treated subgroup. It has been noted that the groups that received PRP treatment presented an increase in the amount of collagen and the granulation tissue and the neo-epidermis was more prominent in the PRP treated rats^54^. A vast number of studies claim the beneficial effect that PRP holds on burn wounds through dermal regeneration and accelerated re-epithelialization^55^.

Studies show that PRP is able to increase the number of hair follicles and the small blood vessels around hair follicles, thus increasing hair density after two to six injections at an interval of a couple of months, with side effects such as scalp sensitivity, mild headaches, pinpoint bleeding, redness and minimal and temporary pain^56^. A number of articles suggest that PRP is a safe tool for treating androgenetic alopecia, which is a type of hair loss that is believed to be caused by genetics^57-59^.

The number of clinical trials regarding the use of PRP in wound healing is increasing year to year, which shows that the need to find a wound healing adjuvant is bigger than anytime. Clinical trials regarding the effect of PRP on wound healing, on acute deep partial thickness thermal injuries, on second and third-degree burns, or on diabetes wounds are not completed. However, there are a few studies that published promising results regarding this topic (see **Table 1**). It is important to mention that being an autologous compound, PRP is not toxic and doesn’t have harmful side-effects. However, in very few cases PRP extractions proved to be ineffective^60^.

**Table 1:** Examples of clinical trials employing PRP use in wound healing

Subject of the study	Number of patients	Results	Source
PRP in reconstructive surgery on children with retractable burn sequelae on extremities	44 (a group received PRP treatment, a group did not receive PRP treatment)	-the wound closure time was not statistically different in the group that received PRP from the group that did not received PRP	ClinicalTrials.gov Identifier: NCT00858442
Effect of PRP and Keratinocyte Suspensions on Wound Healing	45 (a group received standard wound care, a group received PRP treatment, a group received PRP and keratinocyte treatment)	-the groups that received PRP achieved full wound closure faster and presented lower scores for pain than the group that received standard care -the group that received PRP and keratinocyte treatment showed better results than the group that received only PRP treatment	ClinicalTrials.gov Identifies: NCT00856934
Use of PRP and PPP to Prevent Infection and Delayed Wound Healing	515 patients (one group received PRP and PPP treatment, one group did not receive PRP and PPP treatment)	-the groups did not present statistically different results	ClinicalTrials.gov Identifier: NCT01639144

## **6. Conclusions**

Wound healing is a crucial process for tissue homeostasis and for the well-being of the whole organism. Considering the advances made in the field of regenerative medicine, the need to find an adjuvant to aid and speed this process is more and more prominent. Numerous studies have proven that PRP modulates cellular proliferation and migration and tissue regeneration, both in *in vitro *and *in vivo *studies, it is our belief that it can be a useful tool in wound healing. Despite lack of approval by the US Food and Drug Administration (FDA), PRP therapy is gaining unproven acceptance as treatment for sports-related injuries^61,62^. The growth factors found in PRP supply a myriad of properties which showed that are valuable for the re-epithelialization and the regeneration of dermis processes, vital for a proper healing process, as they stimulate the same molecules that are needed in order to cure a lesion.

However, even if there are plenty of studies in scientific literature, larger studies with clinical trials and definite protocols are required, in order to clearly establish PRP’s benefic effects and the exact mechanisms involved in wound healing.

## **KEY POINTS**

**◊** **PRP is a resourceful adjuvant for regenerative processes involved in wound healing**

**◊** **It is widely believed that PRP is one of the most promising auxiliaries in wound healing, since it modulates angiogenesis and cellular proliferation**

**◊** **Large clinical trials with specific preparation methods and rigorous treatments are required in order to clarify the effects of this tool on various wound types.**
